# Socioeconomic and behavioural factors associated with access to and use of Personal Health Records

**DOI:** 10.1186/s12911-020-01383-9

**Published:** 2021-01-13

**Authors:** Ivana Paccoud, Michèle Baumann, Etienne Le Bihan, Benoît Pétré, Mareike Breinbauer, Philip Böhme, Louis Chauvel, Anja K. Leist

**Affiliations:** 1 Department of Social Sciences, Institute for Research on Socio-Economic Inequality, University of Luxembourg, Belval Campus, 4366 Esch-sur-Alzette, Luxembourg; 2 Department of Public Health, University of Liège, Liège, Belgium; 3 Department of General Medicine and Geriatrics, University Medical Centre of the Johannes Gutenberg University Mainz, Mainz, Germany; 4 Department of Endocrinology, Diabetology and Nutrition, Regional Network LORDIAMN, University Hospital of Nancy, Nancy, France

**Keywords:** Personal health records, User acceptance of information technology, Digital divide, Health inequalities, Health inequities

## Abstract

**Background:**

Access to and use of digital technology are more common among people of more advantaged socioeconomic status. These differences might be due to lack of interest, not having physical access or having lower intentions to use this technology. By integrating the digital divide approach and the User Acceptance of Information Technology (UTAUT) model, this study aims to further our understanding of socioeconomic factors and the mechanisms linked to different stages in the use of Personal Health Records (PHR): desire, intentions and physical access to PHR.

**Methods:**

A cross-sectional online and in-person survey was undertaken in the areas of Lorraine (France), Luxembourg, Rhineland-Palatinate and Saarland (Germany), and Wallonia (Belgium). Exploratory factor analysis was performed to group items derived from the UTAUT model. We applied linear and logistic regressions controlling for country-level heterogeneity, health and demographic factors.

**Results:**

A total of 829 individuals aged over 18 completed the questionnaire. Socioeconomic inequalities were present in the access to and use of PHR. Education and income played a significant role in individuals' desire to access their PHR. Being older than 65 years, and migrant, were negatively associated with desire to access PHR. An income gradient was found in having physical access to PHR, while for the subgroup of respondents who expressed desire to have access, higher educational level was positively associated with intentions to regularly use PHR. In fully adjusted models testing the contribution of UTAUT-derived factors, individuals who perceived PHRs to be useful and had the necessary digital skills were more inclined to use their PHR regularly. Social influence, support and lack of anxiety in using technology were strong predictors of regular PHR use.

**Conclusion:**

The findings highlight the importance of considering all stages in PHR use: desire to access, physical access and intention to regularly use PHRs, while paying special attention to migrants and people with less advantaged socioeconomic backgrounds who may feel financial constraints and are not able to exploit the potential of PHRs. As PHR use is expected to come with health benefits, facilitating access and regular use for those less inclined could reduce health inequalities and advance health equity.

## Background

Personal Health Records (PHR) have been championed as a way to improve the access, delivery and the quality of health care services. They are defined as “real-time, patient-centred records that provide immediate and secure information to authorized users” [1]. PHRs are expected to play an increasingly important role in empowering patients by facilitating better health information exchange between patients and health professionals, and in turn enabling patients to be proactive and engage more effectively as partners in their care [2]. It has been noted that the provision of PHRs will further help with self-care, facilitate the better coordination of healthcare services and improve health outcomes [3, 4]. In this context, the European Commission supports the adoption of PHR within and between its member states, with a strong emphasis on the safety and the security of patients’ health data. To date, most countries within the European Union, with the exception of Germany, have developed and to some extent implemented PHRs [1].

However, even though individuals have physical access to their PHRs, the uptake among certain socioeconomic and migrant populations has been rather slow and socially patterned [5–8]. Health inequities might thus be worsened by the fact that technologies that facilitate self-management and patient engagement are used more frequently by those who are already healthier and more socioeconomically advantaged [9, 10]. To date, PHRs have been studied through two different approaches. On one side, scholars are concerned with the digital divide, examining disparities in the use of digital technology across different groups [11–14]. On the other side, research concerned with the use of digital technologies is rooted in the Unified Theory of Acceptance and Use of Technology (UTAUT) approach, predominantly used in the field of social psychology and which explores the individual intentions for the use of Information Communication Technologies (ICT) [15]. The integration of these two approaches can provide a fresh perspective on the ways in which digital technologies may contribute to deepening health inequities.

The notion of the digital divide has been described as a paradigm with two levels. The first level refers to disparities in actual access to digital technology, and the second level goes beyond access and explores the skills and abilities that are required to utilize these technologies [11, 16, 17]. Previous studies have shown that individuals with a higher socioeconomic status are more likely to perform better on both levels of the digital divide. Those with a more advantaged socioeconomic position have a better access to digital technology and also more frequently have the skills required to used them, as compared to individuals from lower socioeconomic strata [7, 12, 18, 19]. Evidence, mainly from the United States, also suggests that racial and ethnic minority patients and those with lower incomes are less likely to have access to and to adopt PHRs [10, 18]. Indeed, it is most likely that those with higher incomes will have earlier access to material goods such as computers, portable health devices or various health monitoring software. Additionally, those with a higher education level are more inclined to use some form of information technology, mostly through their job positions as compared to those from the lower occupational categories whose jobs do not necessarily required contact with ICT.

Van Dijk [16] further distinguishes four broad categories in research on the digital divide: motivational access; physical access; skills and the actual use of digital technologies. He argues that prior to physical access to a digital tool, people need to wish to have access—“*a much neglected phenomenon*” in the digital divide literature [16]. The disengagement with new technologies is explained as involuntary and related to possibilities and lack of opportunities—some people simply do not have access to an ICT or a certain digital technology [20], however, even in places where everyone has access, some people are still not utilizing ICT [21]. This points to the need to look beyond physical access and examine more challenging notions of ‘choice’ and ‘cultural legitimacy’ linked to peoples’ social positions and lifestyles [22]. Indeed, the notion of choice goes back to the sociologist Pierre Bourdieu (1984) who argues that people from more affluent socioeconomic backgrounds make strategic choices that oftentimes lead to a long-term benefit [23]. In the context of the choice as to whether to access their PHRs or not, we can assume that individuals who are more motivated to use this digital tool could exploit its potential and turn it to their health advantage. Conversely, individuals from lower socioeconomic background express a feeling of cultural illegitimacy about using digital devices and generally feel that “the use of ICT oversteps their social position” [24]: p.9]. Although some of Bourdieu’s concepts such as “choice of necessity” and “cultural illegitimacy” has been evidenced in the utilisation of healthcare services and digital self-tracking apps [24, 25] they have not been studied in the field of use and access to PHR. Thus, while the digital divide approach is useful to understand which groups are disadvantaged in the use of new digital technologies and why, it is important to identify specific behavioural processes that lead to individuals’ acceptance and intention to adopt the PHR. This type of approach its best represented by the UTAUT model.

The Unified Theory of Acceptance and Use of Technology (UTAUT) model by Venketesh *et. al.,* 2003, integrates behavioural elements of eight different models and which aims to explain the intention to use digital technologies through six constructs, known as:

1. Performance acceptancy: the degree to which individuals believe that the digital technology will improve their performance;
2. Effort expectancy: the ease of use of the digital technology;
3. Social influence: whether an individual knows someone who uses that technology;
4. Facilitating conditions: the degree of perceived support, such as available help from friends and family in the use of new technology;
5. Personal attitudes towards using digital health technologies;
6. Anxiety: fear of using digital technologies.

Proponents of this theory argue that digital technologies, even if available, are not always accepted by individuals for various reasons, such as: devices that are hard to use, lack of training and computer skills, not seeing the added value in the technology and low social support [26]. However, results show multiple discrepancies in explaining the factors that contribute to the use of digital devices. Hoogenbosch et al*.,* found that effort and performance expectancy were the only constructs that significantly influence patients’ use of a health PHR [27]. Drawing on the UTAUT model, Hoque R and Sorwar G (2017) revealed that, with the exception of facilitating conditions, none of the constructs were associated with the use of a health technology [28]. In addition, researchers that used this model have also argued that the use and the adoption of digital technologies is moderated by demographic variables, especially age and gender [15, 29]. However, literature on the digital divide has shown that there is also a socioeconomic dimension to these disparities that has to be considered.

In this context, the focus of this paper is therefore to integrate the digital divide literature with the UTAUT concepts to provide a better understanding of the socioeconomic and behavioural determinants that contribute to the three stages of PHR use, mainly:

- Expressing a desire to use their PHR;
- Having physical access to their PHR which is achieved through the availability of PHR, as well as a computer and access to the internet, and lastly;
- Intention to regularly use their PHR.

Indeed, as van Dijk [16] highlights that there is a lack of interdisciplinary research, as well as a need to incorporate social psychology into the digital divide research. We believe that UTAUT can shed light on important mechanisms that determine the higher acceptance and use rates among those from more affluent backgrounds. Hence, this study goes beyond the socioeconomic circumstances of individuals by incorporating the UTAUT model.

In particular, we are interested to know:

- Which demographic and socioeconomic factors determine different stages of PHR use: desire to access, physical access and intention to regularly use PHR?
- What behavioural factors linked to the use and acceptance of technology are associated with the intention to regularly use PHR, and are these determined by the socioeconomic characteristics of the individual?

## Methods

### Study design

The study was undertaken as part of a cross-country, collaborative project (INTERREG-APPS) in the Greater Region [30], a cross-border region consisting of the areas of Lorraine (France), the whole of Luxembourg, Rhineland-Palatinate and Saarland (Germany), and Wallonia (Belgium) (Additional file 1: Appendix 1: Map of the Greater Region, licensed under a CC BY 3.0 License). It served also as a tool to raise awareness on the existence of the PHR in the Greater Region (with the exception of Germany where as mentioned above PHRs are not yet available).

A self-administrated questionnaire was developed with a small group of patients’ representatives of each country. The survey was piloted among 24 people across the regions to check completion time and participant comprehension. Following the pilot study, a minor adjustment was made to reflect participant comments. The final version of the survey included questions on demographics, socioeconomic and health status, desire and current access and use of PHR. To measure the main construct ‘intention to use PHR’, we adopted questions from the UTAUT model, which has been validated and empirically tested in a number of studies [15, 27, 28]. After translate and back-translate by native experts, the questionnaire was offered in four different languages: French, German, Luxembourgish and English.

### Participants

The participants (over 18 years of age) were recruited online and in person via various patients’ associations, hospitals and health clinics. In addition to age, participants had to live in one of the Greater Region areas and had to be able to provide consent. Participation in the survey was on a voluntary basis and completely anonymous. Individuals were provided with an information sheet and were informed about the nature of the study, its research aims and its confidentiality policy. Depending on the mode of data collection, a written or an online consent form was obtained from each participant. The study was granted ethical approval by the University of Luxembourg’s Ethics Review Panel.

### Outcome measures

The main three outcome variables in this study were:

1. Having physical access to PHR (“*Do you currently have access to your Electronic Health Record?*”) measured as a binary indicator (yes, no). Those who answered by the negative on this question were directed to the next outcome: whether they had the desire to access their PHR, and those who answered positively were asked to provide further information on their user experience, purpose of access and satisfaction levels.
2. The desire to access PHR (“*Would you like to have access to your Electronic Health Record?”*). If respondents indicated that they do not wish to access their PHR, they were automatically re-directed to the demographic and socioeconomic questions and did not respond to the third outcome of interest—intention to regularly use their PHR. This was done in order to limit missing data.
3. The intention to regularly use their PHR (“*I intend to use my PHR on a regular basis*”) was assessed using a five-point Likert scale from “strongly disagree” to “strongly agree”. This is one of the most used dependent variables in the UTAUT model and a very strong predictor for actual use of digital technologies [31, 32].

### Independent variables

As previous studies show disparities in the use and access to digital health technologies, we used perceived income, education and country of origin as the main socioeconomic status indicators. We also looked at the contribution of individuals’ social networks measured through the number of close relationships with family and friends. When possible, the demographic and socioeconomic questions were drawn from established surveys. Migrants were defined as individuals who were born outside the Greater Region (GR). As educational systems vary across countries, we used the ISCED-2011 educational levels classification to harmonise the educational levels across countries. Household income was measured by self-assessed comfort with participant household income. The question asked respondents to rate their income from: being comfortable on present income; coping on present income; finding it difficult; and finding it very difficult on present income. Despite the wide use of household income as an objective measure of one’s financial situation, it has been argued that subjective income measured through the 'self-assessed comfort with income’ may better capture the financial reality and a wholistic estimate of all components of disposable income that might influence individual attitudes and choices [33]. For example, some individuals with low incomes could still draw on resources from family and friends, which could serve as a buffer to any financial shocks. Next, to assess the intentions of once regular use of PHR we also looked at the association of the technology adoption constructs measured through the six items mentioned above: performance and effort expectancy; social influence; facilitating conditions; anxieties; and personal attitudes towards digital technologies using the UTAUT model by Venkatesh et al. (2003). The survey questions used to measure the UTAUT construct are presented in Additional file 3: Appendix 3: UTAUT items.

### Covariates

A number of confounders were thought to be associated with both access to and intention to use PHR, such as age, gender, employment and partnership status. Previous studies have shown that poor health status and the presence of disease is also associated with the increased probability of using digital health technology [34, 35]. Hence, we also controlled for self-reported chronic disease (yes/no). To account for country-level heterogeneity and differences in the health care systems we accounted for countries-fixed effects [36].

### Data analysis

We used descriptive statistics to analyse the characteristics of the sample. To answer what factors are associated with having access to and the desire to access their PHR, we fitted three logistic regression models. The baseline model included the main effects of the independent variables, the second model included also the covariates (Table 2). In the final model we wanted to understand possible interactions between migration status and perception of income. We therefore introduced two-way interaction terms between these two variables of interest. As confidence intervals of this third model were too large, we report descriptive statistics instead.

For the third dependent variable that measures the degree to which individuals intend to use their PHR, we used linear regressions. Here we also ran three models, one with the UTAUT variables of technology adoption, the second including demographic and socio-economic covariates, and the last one adding an interaction term between education and the UTAUT constructs. However, to facilitate interpretation of the UTAUT six-item components, we utilised explanatory factor analysis with orthogonal rotation to group similar items into broader concepts. Explanatory factor analysis groups together interrelated items in order to reduce the dimensions of variables by clustering items that are highly correlated into a factor which can then be included in the regression analysis [37]. To ensure that the all the variables in the UTAUT construct are correlated in the same direction i.e. positively correlated, before performing the factor analysis we reversed the scale that measured the anxiety levels, and named it lack of anxiety. Internal consistency among the different factors across the UTAUT was tested using Cronbach’s alpha.

## Results

### Sample characteristics

A total of 829 individuals completed the questionnaire. The majority of the sample was female (60%). Its mean age was 44.4 years. While a total of 615 respondents (83%) expressed that they want access to their PHR, 62 respondents (7.5%) said they already have access, and of those only 22 (35%) have already used their PHR. The majority of the respondents were born in one of the countries represented in the Greater Region (87%), with 13% were born outside these four countries. Further participant characteristics are presented in Table 1.

**Table 1:** Description of the sample

	%	N
Gender		
Male	40.07	331
Female	59.93	495
Age		
(mean, SD)	44.4	16.84
Country of residence		
Luxembourg	27.26	226
Belgium	50.78	421
France	14.60	121
Germany	7.36	61
Migration status		
Born in a county of the GR	86.83	646
Not born in a country of the GR	13.17	98
Partnership status		
Living in a partnership	17.88	145
Not living in a partnership	82.12	666
Education		
Primary or less	2.47	20
Secondary	54.81	444
University	42.72	346
Income perception		
Comfortable with their income	39.70	318
Coping on their income	36.70	294
Finding it difficult on their income	17.85	143
Finding it very difficult on their income	5.74	46
Employment status		
Yes	54.23	449
No	14.73	122
Other	31.04	257
Presence of chronic disease		
Yes	36.75	301
No	63.25	518
Want access to their PHR		
Yes	82.55	615
No	17.45	130
Have access to their PHR		
Yes	7.48	62
No	92.52	767
Intend to use PHR regularly		
(Mean, SD)	3.52	1.03

### Results from the exploratory factor analysis

The factor analysis pointed to the existence of three key dimensions among the eight questions asked to represent the six dimensions of the UTAUT. The first factor grouped the perceived usefulness and ease of use in one common factor, the second factor captured individuals’ social influence and the support of family and friends in the use of PHR, and the last one grouped the two items that measure an individual’s anxiousness in the use of digital technology. The table with the detailed results of the factor analysis can be found in Additional file 2: Appendix 2: Factor loadings of the ‘Intention to use PHR’ items. Cronbach’s alpha coefficient confirmed the reliability of the factors (α = 0.79), with the set of items being closely related with a relatively high internal consistency. The three factors were used instead of the eight answers to UTAUT-related questions in the regression analysis.

### Multivariate regression

Table 2 shows the results from the fully adjusted models, associations of the demographic and socioeconomic factors with whether individuals want to access their PHR at all, and the likelihood of respondents reporting physical access to their PHR. The results of the model which included interaction term between migration status and perceived income are not shown due to the large confidence intervals, therefore we considered the model without interaction terms to be more parsimonious. However, below we present the relevant descriptive statistics to explore this question.

**Table 2:** Logistic regression, desire to access and physical access to PHR in the Greater Region

Outcomes	Desire to access their PHR		Have physical access to PHR	
	OR	95% CI	OR	95% CI
Country *(Ref. group: Belgium)*				
Germany	0.915	(0.319, 2.623)		
France	1.746	(0.676, 4.51)	4.444	(0.157, 12.57)
Luxembourg	3.014***	**(1.557, 5.834)**	1.263	(0.554, 2.878)
Gender *(Ref. group: Male)*				
Female	2.139***	**(1.357, 3.372)**	0.705	(0.342, 1.451)
Age *(Ref. group: 18–15)*				
36–65	0.911	(0.525, 1.581)	1.784	(0.717, 4.443)
Over 65	0.487*	**(0.240, 0.987)**	3.000	(0.873, 10.31)
Partnership status *(Ref. group: not living in a couple)*				
Living in a couple	1.214	(0.578, 2.547)	1.178	(0.453, 3.066)
Migration status *(Ref. group: born in the Greater Region)*				
Not born in the Greater Region	0.430***	**(0.230, 0.806)**	2.587*	**(1.087, 6.158)**
Employment status *(Ref. group: employed)*				
Not employed	0.825	(0.406, 1.68)	0.561	(0.153, 2.058)
Chronic disease *(Ref. group: Living with a chronic disease)*				
No	0.984	(0.606, 1.597)	1.330	(0.604, 2.928)
Perception of income *(Ref. group: difficult/very difficult)*				
Coping/comfortable	1.866**	**(1.112, 3.129)**	2.561*	**(1.060, 6.188)**
Education *(Ref. group: secondary and less)*				
University and above	2.351***	**(1.364, 4.052)**	1.267	(0.592, 2.712)
Social network	1025	(0.918, 1.146)	0.959	(0.570, 1.074)

Bold values indicate *P* < 0.05

*P* < 0.10; *P < 0.05; **P < 0.01; ***P < 0.001

### Desire to access PHR

After controlling for country fixed effects and demographic variables, the results indicate that a higher educational level (OR = 2.35, 95% CI = 1.36–4.05) and living comfortably on income (OR = 1.87, 95% CI = 1.11–3.13) are positively associated with the desire to have access to their PHR in the expected direction. However, being over the age of 65, and being a migrant, i.e., born outside of the Greater Region, was negatively associated to the desire to access their PHR. Women were more likely to report the desire to access their PHR (OR = 2.14, 95% CI = 1.36–3.37). In this sample, the presence of a chronic disease, partnership status and number of individuals in one’s social network were not associated with having a desire to access PHR.

Our descriptive analysis of the migrants with different financial constrains revealed that compared to 16 percent of non-migrants, 24 percent of migrants reported that they do not wish to access their PHR. Of migrants who do not wish to access their PHR, the majority (71%) stated that it is difficult or very difficult to live on their present income. On the other hand, 76 percent of those migrants who wish to have access to their PHR stated that they are comfortable or coping on their present income.

### Having physical access to PHR

Exploring the association between those who have physical access to their PHR and the different demographic and socioeconomic variables, we found that those born outside of the Greater Region and those who reported that they are living comfortably or coping on present income were more likely to have physical access to their PHR (OR = 2.59, 95% CI = 1.087–6.158), (OR = 2.56, 95% CI = 1.060–6.188), respectively.

### Intention to regularly use PHR

In the first step, we explored the contribution of demographic and socioeconomic variables to the intention to use PHR. The first model, adjusted for country-fixed effects, shows a clear gradient when considering regular use of PHR: higher educational level was positively associated with the intention to regularly use (Table 3). After additionally including the UTAUT-derived factors in Model 2, education was rendered insignificant. However, we found a strong association between the UTAUT constructs and the intention to regularly use PHR. Individuals who perceive the PHR to be useful and those who have the necessary digital skills were more inclined to use the PHR regularly. The results further demonstrated that social influence and support and lack of anxiety in using technology were strong predictors of the regular use of medical records. As we were particularly interested in the association of the UTAUT behavioural variables with socioeconomic factors we also modelled an interaction between the education level and the UTAUT factors (not presented), however the pairwise interaction was not significant, and therefore not included in the model.

**Table 3:** OLS regression, association between intention to regularly use PHR and demographic, socioeconomic and UTAUT factors

	Model 1		Model 2	
	Coef	95% CI	Coef	95% CI
Country *(Ref. group: Belgium)*				
Germany	0.130	(−0.241, 0.502)	0.174	(−0.268, 0.615)
France	0.172	(−0.086, 0.43)	0.151	(−0.086, 0.388)
Luxembourg	0.429	(0.196, 0.662)	0.394	(0.202, 0.586)
Gender *(Reference group: Male)*				
Female	0.140	(−0.041, 0.321)	0.098	(−0.066, 0.261)
Age	0.005	(−0.001, 0.011)	0.004	(−0.001, 0.009)
Partnership status *(Ref. group: not living in a couple)*				
Living in a couple	0.036	(−0.208, 0.281)	0.002	(−0.214, 0.216)
Migration status *(Ref. group: born in the Greater Region)*				
Not born in the Greater Region	−0.189	(−0.494, 0.115)	0.023	(−0.260, 0.305)
Employment status *(Ref. group: employed)*				
Not employed	−0.026	(−0.271, 0.219)	−0.023	(−0.224, 0.178)
Chronic disease *(Ref. group: Living with a chronic disease)*				
No	−0.174	(−0.369, 0.021)	−0.225***	**(**−**0.387, **−**0.063)**
Perception of income *(Ref. group: difficult/very difficult)*				
Coping/comfortable	0.211	(−0.002, 0.423)	−0.011	(−0.213, 0.191)
Education *(Ref. group: secondary and less)*				
University and above	0.181*	**(0.007, 0.356)**	0.092	(−0.06, 0.245)
Use of Technology				
PHR useful & easy			0.550***	**(0.456, 0.645)**
Social influence & support			0.123***	**(0.032, 0.213)**
Lack of anxiety			0.204***	**(0.116, 0.292)**

Bold values indicate *P* < 0.05

*P* < 0.10; *P < 0.05; **P < 0.01; ***P < 0.001

## Discussion

This study contributes to the literature on PHR access and adoption in two ways. The study explores three different stages in the adoption of PHR, mainly desire to access, physical access and intention to regularly use PHRs, and integrates both socioeconomic and technology related factors. The findings suggest that although closely related, each of the three stages of PHR use is determined by different factors. While education plays a larger role in the desire to have access to PHR, the effect of subjective income operates through the possession of the material factors needed to have physical access to PHR. On the other hand, for respondents’ intentions to regularly use their PHR, socioeconomic factors were supplemented by the perceived usefulness and ease of navigation of the PHR, as well by an individual’s level of social influence and support from family and friends. Some of the main findings is that there is a clear gradient in the desire to access PHRs and in having physical access, with those more educated and living comfortably on present income showing a stronger desire to obtain access to their PHR. This is in line with previous research indicating that those from lower socioeconomic backgrounds show lack of interest in digital devices [24, 37]. Migrants and those living more comfortably on present income are more likely to have physical access to their PHR compared to those living less comfortably. As some studies highlight this might be linked to the ability to own technologies or broadband internet, or a higher awareness of their existence [10, 16, 39]. Migrants may be also more likely to possess digital devices and internet as a mean of communication with their families and friends in the country of origin. However, when it comes to the desire to access PHR, the results demonstrate that being a migrant, male and of older age is negatively associated with the desire to access PHRs. Some scholars point out, that the desire to access to PHR might be linked to the issue of trust in health professionals, anxieties and technophobia [9, 16, 40]. Stronger anxiety, feelings of cultural illegitimacy, reluctance or even rejection of ICT has been also shown to act as a barrier of use and adoption of health digital technologies among the lower socioeconomic cohorts [24].

The study further highlights the importance of perceived income among migrants when looking at issues of desire to access PHR. Income comfort among migrants was an important determinant of whether they want to access their PHRs, with those feeling in a difficult financial situation not wishing to have access to PHRs at all. On the contrary, migrants who are feeling more comfortable with their income were more likely to want to have access and to have physical access to their PHR, which shows the disadvantages faced by migrants with a lower perceived income. Drawing on Bourdieu’s theory of practice, and his concept of ‘choice of necessities’ this could be explained through the fact that peoples’ lifestyle choices and attitudes are based on their socioeconomic circumstances [23]. Those who are free of economic necessities are able to make long-term choices that are independent of their day-to-day circumstances. In this context, it could be argued that those who feel more financially comfortable can make more long-term strategic choices such as having access to their PHR in order to better manage their health. Therefore, health professionals play a vital role in showing the benefits of the PHR and in offering encouragement to individual to obtain access to their records. An alternative possibility would be to provide universal PHR access on an opt-out basis, with a possibility to close or permanently delete the PHR at any time. At the moment, individuals who are aware of the existence of PHRs can voluntarily access or they are invited to access their records by their clinicians. However, as evidenced by Ancker et al. 2017, an opt-in policy of access to patient PHRs was associated with socioeconomic disparities [10]. Of course, for this policy to be fruitful more structural factors have to be addressed such as access to a computer and the internet.

On the other hand, our results demonstrate that UTAUT provides a useful framework to uncover potential mechanisms through which individuals intend to use their PHR on a regular basis. In this sample, perceived usefulness and ease of use were the strongest predictors of PHR acceptance and use, followed by individual’s social influence and support and anxieties related to the use of new technology or data security. Although in the first model we found that education was associated with the regular use of PHR, in the model where we included the UTAUT-derived factors, education lost its significance. This results are somewhat surprising as in the digital divide literature it has been noted that technical competence and digital literacy is a strong factor that influences the use of technology [11, 16]. However, we assume that with the current regression model it is difficult to disentangle the effect of education and the UTAUT derived factors. More complex mediation analysis is required to uncover the exact mechanisms and pathways through which socioeconomic factors play role in the specific UTAUT constructs. Finally, our findings confirmed results of other studies and showed the presence of chronic disease is closely associate with the individuals use of PHR.

Although this study is based on a unique harmonised cross-country design, given the limitation of the sample and the nature of the convenience sampling technique, there could be selection bias due to selective enrolment into the project. Although in-person survey promotion was undertaken in some countries, the answers might be biased towards those who already have access to a computer or the internet. However, prevalence of PHR use in Luxembourg in this study was in line with the prevalence of use in the general population. A comparison of PHR users in this study with PHR users in the general population of the four involved countries is unfortunately not possible due to a lack of registries. According to Eurostat in 2019, in all four countries of the region 90% of the adult population reported having used the internet on a daily basis, and more than 80% have used some form of ICT [41]. Lastly, we were not able to undertake regional-level comparisons, given that the sample sizes for Germany (n = 61) and France (n = 121) were insufficient to make statistical inferences. In addition, we also had a small number of patients who actually used their PHR (n = 22). It was thus not possible to undertake multivariate statistical analysis on the characteristics of these participants, and on whether there are any socioeconomic differences in the purposes for which individuals used their PHR, and to fully understand the digital divide phenomenon. With increasing prevalence of use in the general population, it is important that further studies provide insights into this particular facet of PHR use.

## Conclusion

Our study highlights the importance of considering all stages in the use of PHR. If PHR are to be implemented successfully and among all socioeconomic groups, policy-makers need to take into consideration each stage of PHR use: desire to use PHR, make sure everyone is aware and has a physical access to PHR, and encourage adoption and regular use of PHR, though designing and promoting user-friendly records which are easy to navigate. Availability of PHR is not sufficient as such and must come along with appropriate training of individuals from less advantaged socioeconomic backgrounds. At the same time health professionals need to explain the added value of the PHR to their patients. Special attention needs to be paid to those who are not motivated or who do not wish to have access to their PHR. As our results demonstrated, these are the most disadvantaged groups who may not be able to grasp the benefits they could derive from the regular use of their PHR. Given this, it is paramount to understand and address more structural factors such as individuals’ feelings of financial constraints that may shape peoples’ choices and practices. Failing to do so could exacerbate already existing health inequities.

## Supplementary Information

**Additional file 1:** Map of the Greater Region is appropriate.

**Additional file 2:** Factor loadings of the ‘Intention to use PHR’ items.

**Additional file 3:** UTAUT items.

## Data Availability

The dataset supporting the conclusions of this article is available from the corresponding author upon reasonable request.

## Abbreviations

PHR: Personal Health Record
ICT: Information Communication Technologies
UTAUT: User Acceptance of Information Technology
INTERREG: Interregional
APPS: Approche Patient Partenaire de Soins
GR: Greater Region
