# Supplementary material for: Socioeconomic and behavioural factors associated with access to and use of Personal Health Records
Source: BMC Med Inform Decis Mak. 2021 Jan 13;21:18. doi: 10.1186/s12911-020-01383-9 (PMC7805047; doi:10.1186/s12911-020-01383-9)
Supplement: Supplementary file 1 — Additional file 1: Map of the Greater Region is appropriate. [file 12911_2020_1383_MOESM1_ESM.docx]

Appendix 1: Map of the Greater Region


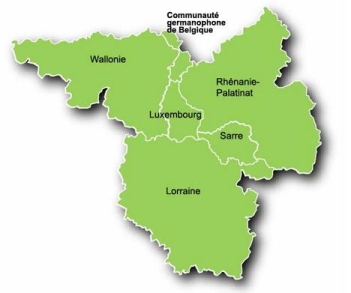


Legend. Source: Luxembourg for ICT (2009), Accessed on Wikimedia Commons (CC BY 3.0)
