# Supplementary material for: Socioeconomic and behavioural factors associated with access to and use of Personal Health Records
Source: BMC Med Inform Decis Mak. 2021 Jan 13;21:18. doi: 10.1186/s12911-020-01383-9 (PMC7805047; doi:10.1186/s12911-020-01383-9)
Supplement: Supplementary file 2 — Additional file 2: Factor loadings of the ‘Intention to use PHR’ items. [file 12911_2020_1383_MOESM2_ESM.docx]

**Socioeconomic and behavioural factors associated with access to and use of Personal Health Records**

**Appendix 2. Factor loadings of the Intention to use PHR items**

| *Factor analysis of Intention to use PHR items* |  |  |  |
| --- | --- | --- | --- |
| **ITEM** | Factor 1 | Factor 2 | Factor 3 |
| **Factor name** | PHR useful & easy | Social support | Lack of anxiety |
|  |  |  |  |
| 1. I think using my PHR would be a useful tool in managing my own-health | **0.8637** | -0.0148 | -0.0313 |
| 2. I think It will be easy to use my PHR | **0.8623** | -0.1023 | -0.0029 |
| 3. I have the digital knowledge necessary to use my PHR | **0.7687** | -0.0623 | 0.1938 |
| 4. I think that using my PHR is a good idea | **0.6054** | -0.1859 | 0.1953 |
| 5. My family and friends are using PHR | -0.0876 | **0.8573** | -0.0238 |
| 6. I have someone to explain to me how to use my PHR | -0.1361 | **0.8481** | 0.0714 |
| 7. I am hesitant to use my PHR for fear of exposing my health information | 0.0031 | -0.0874 | **0.8306** |
| 8. I feel nervous about using my PHR | 0.1995 | 0.1452 | **0.7253** |
