# Supplementary material for: Socioeconomic and behavioural factors associated with access to and use of Personal Health Records
Source: BMC Med Inform Decis Mak. 2021 Jan 13;21:18. doi: 10.1186/s12911-020-01383-9 (PMC7805047; doi:10.1186/s12911-020-01383-9)
Supplement: Supplementary file 3 — Additional file 3: UTAUT items. [file 12911_2020_1383_MOESM3_ESM.docx]

**Socioeconomic and behavioural factors associated with access to and use of Personal Health Records**

**Appendix 3. UTAUT survey questions**

| 1. *Please indicate whether you agree or disagree with the following statements* | | | | | |
| --- | --- | --- | --- | --- | --- |
|  | Strongly  disagree | Disagree | Neither agree or disagree | Agree | Strongly  Di agree |
| 1. I think using my PHR would be a useful tool in managing my own-health | ❑ | ❑ | ❑ | ❑ | ❑ |
| 2. I think It will be easy to use my PHR | ❑ | ❑ | ❑ | ❑ | ❑ |
| 3. I have the digital knowledge necessary to use my PHR | ❑ | ❑ | ❑ | ❑ | ❑ |
| 4. I think that using my PHR is a good idea | ❑ | ❑ | ❑ | ❑ | ❑ |
| 5. My family and friends are using PHR | ❑ | ❑ | ❑ | ❑ | ❑ |
| 6. I have someone to explain to me how to use my PHR | ❑ | ❑ | ❑ | ❑ | ❑ |
| 7. I am hesitant to use my PHR for fear of exposing my health information | ❑ | ❑ | ❑ | ❑ | ❑ |
| 8. I feel nervous about using my PHR | ❑ | ❑ | ❑ | ❑ | ❑ |
| 9. I intent to use my PHR on a regular basis | ❑ | ❑ | ❑ | ❑ | ❑ |
